# Downhill, Ultrafast and Fast Folding Proteins Revised

**DOI:** 10.3390/ijms21207632

**Published:** 2020-10-15

**Authors:** Mateusz Banach, Katarzyna Stapor, Leszek Konieczny, Piotr Fabian, Irena Roterman

**Affiliations:** 1 Department of Bioinformatics and Telemedicine, Jagiellonian University—Medical College, Lazarza 16, 31-533 Krakow, Poland; mateusz.banach@uj.edu.pl; 2 Institute of Computer Science, Silesian University of Technology, Akademicka 16, 44-100 Gliwice, Poland; katarzyna.stapor@polsl.pl (K.S.); piotr.fabian@polsl.pl (P.F.); 3 Chair of Medical Biochemistry, Medical College, Jagiellonian University, Kopernika 7, 31-034 Krakow, Poland; mbkoniec@cyf-kr.edu.pl

**Keywords:** ultrafast folding, downhill folding, hydrophobic core

## Abstract

Research on the protein folding problem differentiates the protein folding process with respect to the duration of this process. The current structure encoded in sequence dogma seems to be clearly justified, especially in the case of proteins referred to as fast-folding, ultra-fast-folding or downhill. In the present work, an attempt to determine the characteristics of this group of proteins using fuzzy oil drop model is undertaken. According to the fuzzy oil drop model, a protein is a specific micelle composed of bi-polar molecules such as amino acids. Protein folding is regarded as a spherical micelle formation process. The presence of covalent peptide bonds between amino acids eliminates the possibility of free mutual arrangement of neighbors. An example would be the construction of co-micelles composed of more than one type of bipolar molecules. In the case of fast folding proteins, the amino acid sequence represents the optimal bipolarity system to generate a spherical micelle. In order to achieve the native form, it is enough to have an external force field provided by the water environment which directs the folding process towards the generation of a centric hydrophobic core. The influence of the external field can be expressed using the 3D Gaussian function which is a mathematical model of the folding process orientation towards the concentration of hydrophobic residues in the center with polar residues exposed on the surface. The set of proteins under study reveals a hydrophobicity distribution compatible with a 3D Gaussian distribution, taken as representing an idealized micelle-like distribution. The structure of the present hydrophobic core is also discussed in relation to the distribution of hydrophobic residues in a partially unfolded form.

## 1. Introduction

Despite many years of research [1], the mechanism of the protein folding process remains unresolved. The protein folding process turns out to be varied, including the time it takes to obtain the native structure. There are proteins with a small number of amino acids in the chain which are characterized by a very fast folding process. This group of proteins is known as fast-folding, ultrafast folding or downhill [2].

Another group of proteins are those that fold on their own, although the participation of the ribosome is required [3]. An example of an extremely demanding process is the folding process which requires the participation of chaperones or chaperonins [4,5,6,7,8].

When discussing the issue of protein folding, the presence of many intrinsically disordered proteins should also be noted [9]. Generally speaking, in statistical terms, the course of the folding process should last a very long time [10]. However, this is not so. Fast-folding proteins reach their final structural form within a dozen microseconds [11]. The identified multi-stage nature of the folding process introduces the term intermediates, the number of which is characteristic for a given protein. [12,13,14,15]. An often identified state of partial unfolding is referred to as the molten globule state [16,17].

New perspectives are emerging with the appearance of techniques based on the “single molecule” experiment, including nuclear magnetic resonance, relaxation dispersion NMR spectroscopy [18] (in particular, photo-protection strategy [19]), as well as single molecule fluorescence spectroscopy sub-millisecond conformational dynamics [20]. Single molecule fluorescence resonance energy transfer (SM-FRET) utilizing fluorescent labeling and immobilization of proteins opens the possibility of quantitative (temporal) analysis of the folding process for fast-folding proteins [21].

Among the possible mechanisms of the protein folding process, hydrophobic collapse is considered [22,23]. Thermodynamic analysis even suggests that the smallest proteins are expected to have very marginal free energy barriers to folding [24,25,26,27]. The presence of specific secondary forms, including the Beta-hair pin, is important in obtaining the final structure of the protein [28,29].

A list of widely used experimental techniques is provided in a review [30]. The current analysis takes into account the presence of the water environment in folding the protein structure. The influence of the water environment on the structure of proteins as described by the fuzzy oil drop model has already been discussed many times, including structures with distribution expressed by the 3D Gaussian function and structures showing local incompatibility both in the form of local excess [31] and deficit hydrophobicity [32].

The conclusion drawn from these observations indicates that there are sequences that are capable of generating structures with a highly ordered hydrophobic core. Local non-compatibility with the idealized distribution can even be treated as intentional. This imperfection is a form of encoding the biological function of a given protein. Proteins with micelle-like hydrophobicity distribution are very soluble, but completely lack interaction opportunities with other molecules, except for surface complexing of ions or low-molecular compounds on the basis of charge interaction. Proteins with a high degree of similarity in the hydrophobicity distribution to the idealized one would be devoid of their biological activity. It turns out, however, that the biological function of proteins with micelle-like distribution is of biological importance in the case of antifreeze type III proteins, where high solubility is desirable and where binding or complexation of other molecules is not expected [33].

The aim of the presented analysis is to propose a folding mechanism for proteins characterized by a fast-folding process based on the active participation of the water environment in the generation of a hydrophobic core. This directs the process towards the concentration of hydrophobic residues in the center with simultaneous exposure of polar residues on the surface [34,35,36,37].

To demonstrate an additional factor promoting rapid reach of the native structure, partially unfolded structures based on the early-stage model have also been included in the analysis [38,39,40]. Partially unfolded structures that retain the fragmentary concentration of hydrophobic residues and contain a seed for the construction of a hydrophobic core favor a high prognosis for a reversible process.

## 2. Results

As previously mentioned, the potential high rate of folding of the polypeptide chain was considered by evaluating the degree of generation of the hydrophobic core, in which the water environment was actively involved as the dominant factor. The hydrophobic core was regarded as an important factor stabilizing the tertiary structure (apart from the disulfide bonds). According to the fuzzy oil drop model, the term “presence of a hydrophobic core” means both the concentration of high hydrophobicity in the center of the molecule together with the presence of a polar surface coating.

**Figure 1:**
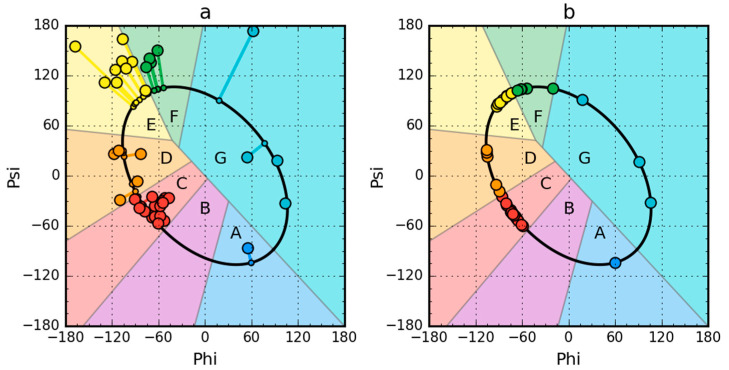
Model of a partially unfolded structure for peripheral-subunit binding domain (PDB ID: 1W4E) protein used as example. (**a**) Phi, Psi angles distribution in the structure available in PDB transformed to the appropriate Phi_e_ and Psi_e_ angles (shortest distance criterion). (**b**) Status after transformation and optimization. The colors distinguish the zones. A–G are defined according to seven local maxima on the ellipse path (limited conformational sub-space). The ellipse path defined according to Phi, Psi change causing the change of radius of curvature of the polypeptide.

The status expressed by the values of RD (Relative Distance) parameters for both the native and partially unfolded forms is given in Table 1. Figure 1 presents the idea of obtaining a partially unfolded form generated based on the Phi_e_ and Psi_e_ angles.

Partially unfolded structures for all the proteins under study were obtained as shown in Figure 1.

The proteins presented in Table 1 show RD values below 0.5 in the vast majority for the native structures available in PDB. The structure of the e3-binding domain (PDB ID: 2CYU) for the complete structure shows an RD greater than 0.5. However, this value is due to loose loops. The system of three helices constituting the main part of the whole molecule shows RD = 0.493, which indicates the formation of a hydrophobic concentration in the center of the molecule.

The molecule 1BBL, also showing an RD above 0.5, reduces the value to 0.462 when the four residues in the loop are eliminated. Moreover, the status of two components of the whole molecule composed of helical fragments 12–33 and 38–48 (neglecting the loose loop) indicates a very high adjustment of the hydrophobicity distribution to the micellar system (both values below 0.4).

The hypothesis that the active participation of the water environment in protein folding, in which the formation of the hydrophobic core is important, has been demonstrated.

### 2.1. Peripheral-Subunit Binding Domain (1W4E)

**Figure 2:**
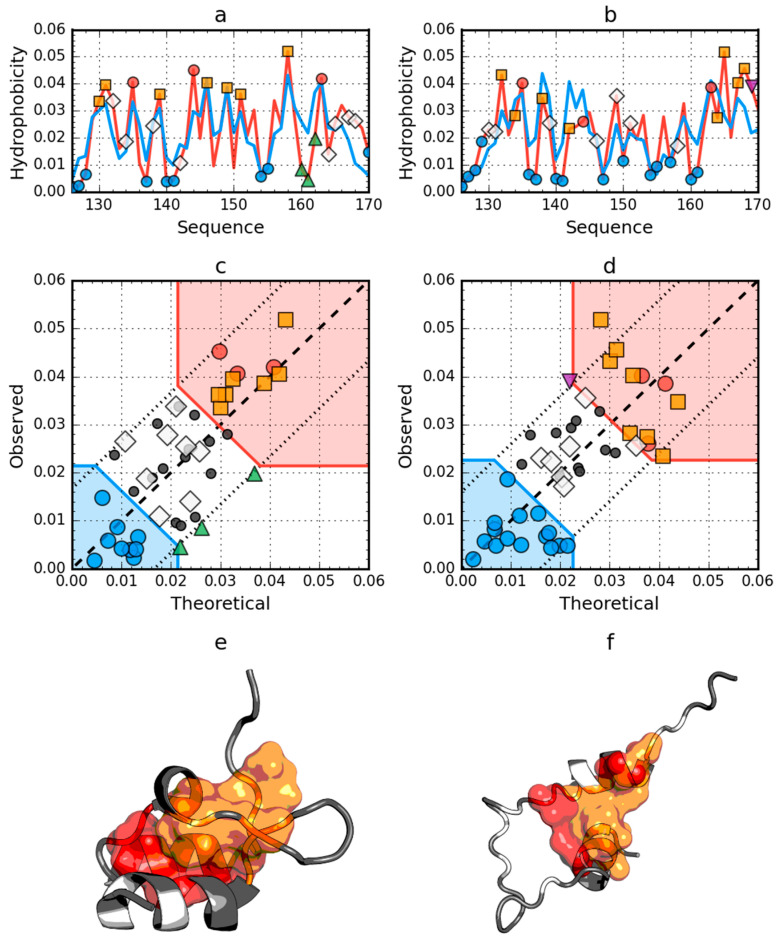
Hydrophobicity density distributions and 3D presentation of native (**a**,**c**,**e**) and early stage (**b**,**d**,**f**) forms of peripheral-subunit binding domain (PDB ID: 1W4E). Blue line on (**a**,**b**): theoretical distribution (*T*); red line on (**a**,**b**): observed distribution (*O*); red zone on (**c**,**d**): hydrophobic core; blue zone on (**c**,**d**): hydrophilic surface; red circle markers: common hydrophobic core members in both models; orange square markers: hydrophobic core members in this model but not in the other model; white rhombus markers: hydrophobic core members in other model but not in this model (possible overlap with other markers); blue circle markers: hydrophilic surface members in this model; green triangle markers: *T* value >> *O* value; purple triangle markers: *T* value << *O* value: black circle markers: hydrophobically insignificant residues. Colored segments on (**e**,**f**) correspond to markers of the same color on (**a**–**d**), respectively (hydrophobic core is shown as surface).

The peripheral-subunit binding domain (PDB ID: 1W4E) represents a set of models with an identical sequence. The status of both the native and partially unfolded form shows the presence of a high degree of arrangement compatible with the fuzzy oil drop model. In this case, the reconstruction of a structure with a high degree of packing which satisfies the conditions resulting from this model, is obvious (Figure 2).

### 2.2. W Protein of Bacteriophage Lambda (2L6Q)

**Figure 3:**
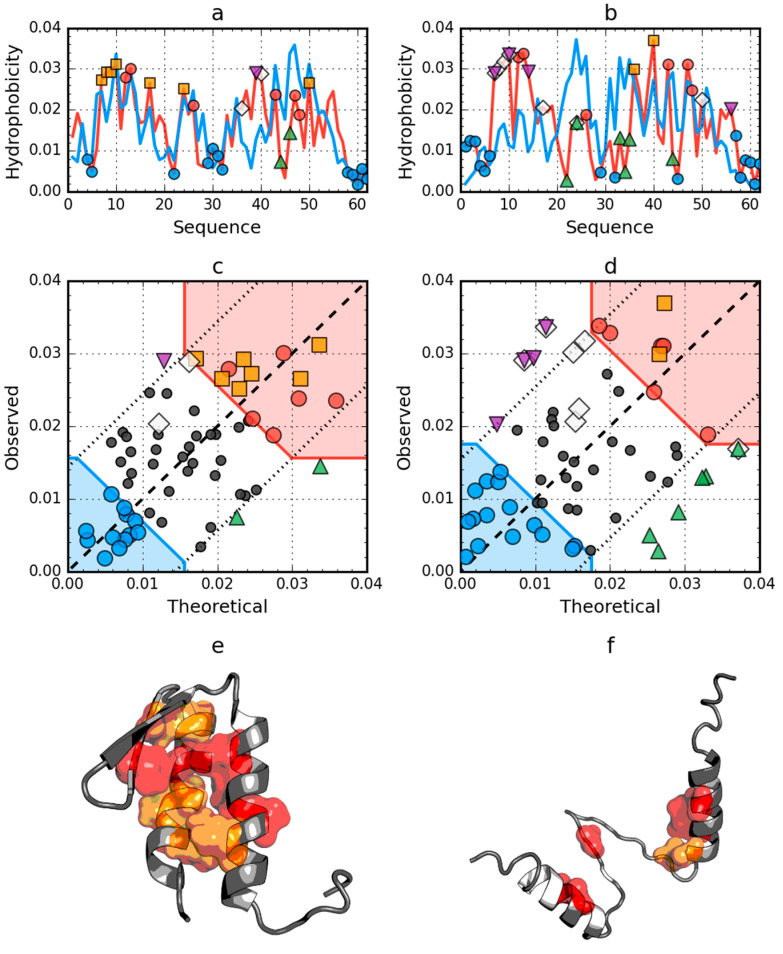
Hydrophobicity density distributions and 3D presentation of native (**a**,**c**,**e**) and early stage (**b**,**d**,**f**) forms of W protein of bacteriophage lambda (PDB ID: 2L6Q). Blue line on (**a**,**b**): theoretical distribution (*T*); red line on (**a**,**b**): observed distribution (*O*); red zone on (**c**,**d**): hydrophobic core; blue zone on (**c**,**d**): hydrophilic surface; red circle markers: common hydrophobic core members in both models; orange square markers: hydrophobic core members in this model but not in the other model. White rhombus markers: hydrophobic core members in other model but not in this model (possible overlap with other markers); blue circle markers: hydrophilic surface members in this model; green triangle markers: *T* value >> *O* value; purple triangle markers: *T* value << *O* value; black circle markers: hydrophobically insignificant residues. Colored segments on (**e**,**f**) correspond to markers of the same color on (**a**–**d**), respectively (hydrophobic core is shown as surface).

The structure of W protein of bacteriophage lambda (PDB ID: 2L6Q) shows the presence of an arranged core in its native form (Figure 3 and Table 1). In the 2L6R, the presence of the fuzzy oil drop model arrangement was also demonstrated in a partially unfolded form. In the case of the unfolded 2L6Q, only the segment Beta-strand 22–28 shows a local distribution different from the expected one. The low RD value for the native form, however, suggests that the process of hydrophobic core generation gives this segment a status compatible with the idealized distribution (Figure 3a).

### 2.3. Other Proteins

The analysis of two further sample proteins (thermostable protein variant engrailed homeodomain (PDB ID: 2P6J) and de novo designed triple helix bundle protein (PDB ID: 2A3D)) are included in Supplementary Materials (Figures S1 and S2) to avoid redundancy.

### 2.4. Comparative Analysis of Partially Unfolded Structures

**Figure 4:**
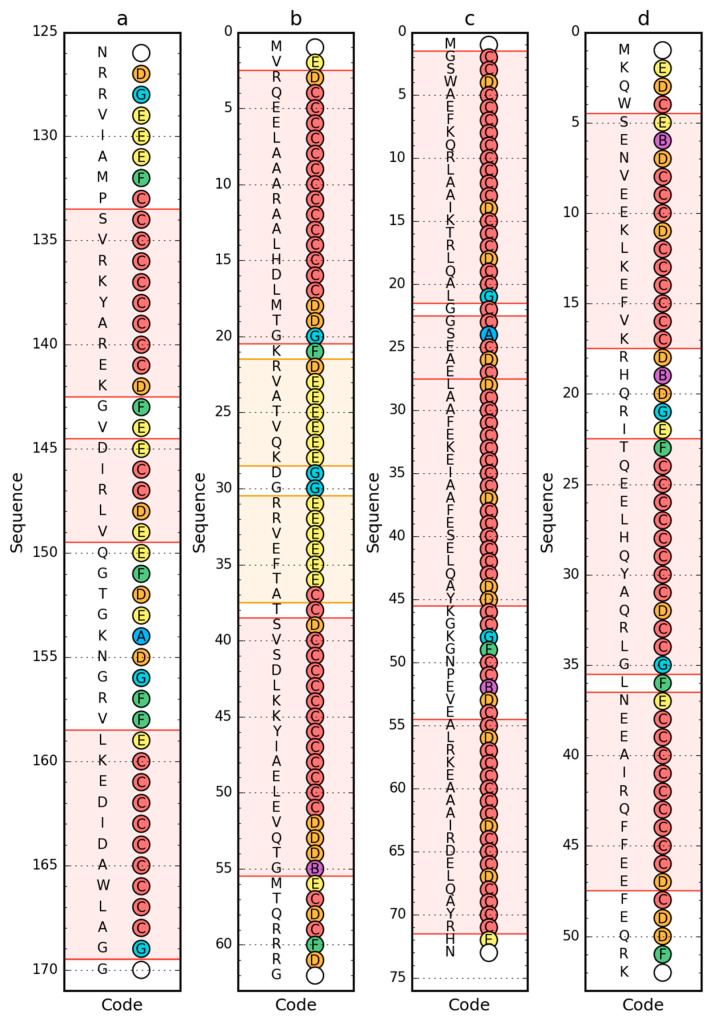
Sequence (left) and structural codes (right) of peripheral-subunit binding domain (PDB ID: 1W4E) (**a**); W protein of bacteriophage lambda (PDB ID: 2L6Q) (**b**); de novo designed triple helix bundle protein (PDB ID: 2A3D) (**c**); and thermostable protein variant, engrailed homeodomain (PDB ID: 2P6J) (**d**). Colors of the circle markers correspond to structural codes (A: blue, B: purple, C: red, D: orange, E: yellow, F: green, G: cyan). Chain termini are marked by blank circles. The frames are as follows: red, helical form (code C expected); yellow/orange, β-structural (code E expected).

Using a set of structural codes (A–G) where C stands for the right-handed helix, E stands for the beta structure and G represents the left-handed helix, the presence of these forms of the secondary structure of the proteins in question was determined (Figure 4). The D code, which expresses a structural form intermediate between the helix and the beta form, is also worth mentioning. Also important is the F code, which represents the forms present at the end of the beta strand, introducing a significant reduction in the radius of curvature in relation to the large radius values for the beta-strand form.

**Figure 5:**
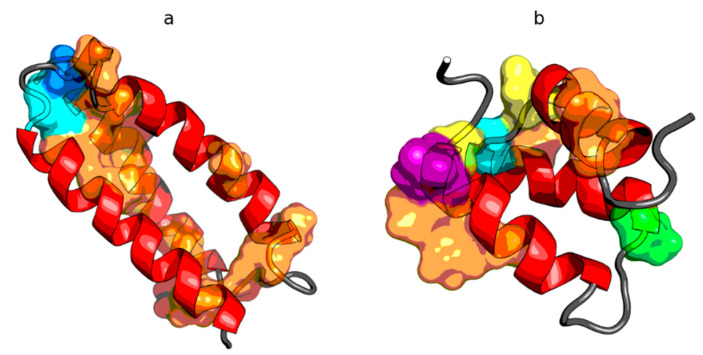
3D presentation of de novo designed triple helix bundle protein (PDB ID: 2A3D) (**a**) and engrailed homeodomain (PDB ID: 2P6J) (**b**) with localization of residues representing structural codes other than the expected C within the helical fragments. These discrepancies are shown as the surface, with colors matching the color codes in Figure 4c or Figure 4d respectively (the helices themselves are red, just like code C is represented by red on Figure 4).

The form of secondary structure as classified by PDB [40] with PROMOTIF [41] turns out to be different to those determined by structural codes (Figure 5). This is especially true of engrailed homeodomain (PDB ID: 2P6J), where in helical fragments 5–17 and 23–35 are also defined by structural codes other than C. In other cases, the codes differing from the classified form occur only for the N- and C-terminal residues in individual segments. This is an irrelevant difference from the point of view of determining the status of a given residue in the context of the accompanying segment. However, it strongly influences the formation of ES structural form.

### 2.5. Negative Control

**Figure 6:**
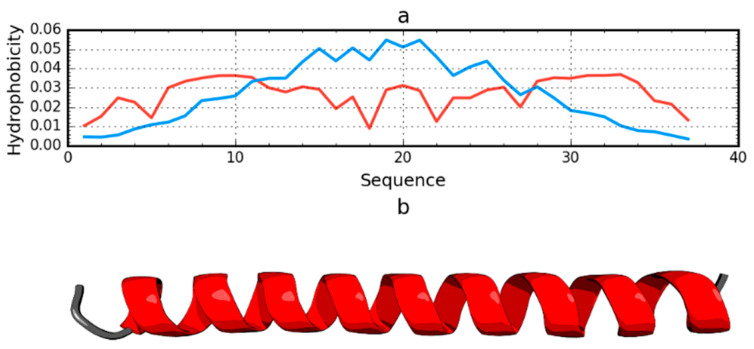
Hydrophobicity density distributions (**a**) and 3D presentation (**b**) of antifreeze protein from winter flounder (PDB ID: 1WFA). Blue line: theoretical distribution (*T*); red line: observed distribution (*O*).

Among the proteins acting as an anti-freeze agent is the winter flounder derived protein (PDB ID: 1WFA). This short polypeptide chain (37 aa) does not generate a globular form; instead, it takes the form of a helix that spans the entire chain. This form is unable to generate a hydrophobic core. The use of the fuzzy oil drop model unanimously reveals the status of this protein as not representing a micelle-like form. The RD value for this protein is 0.837. The *T* and *O* profiles illustrating the hydrophobicity distribution show the expected hydrophobicity concentration, which was not observed (Figure 6). There was also no significant reduction in the level of observed hydrophobicity as one approached the terminal positions of the amino acids.

**Figure 7:**
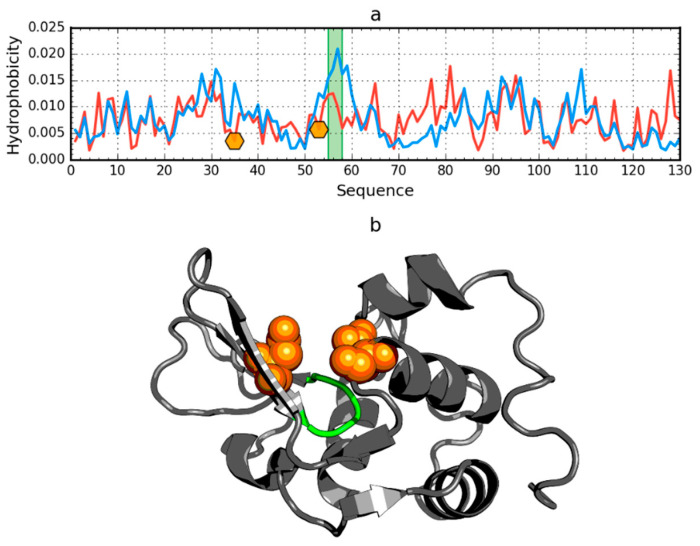
Hydrophobicity density distributions (**a**) and 3D presentation (**b**) of human lysozyme (PDB ID: 1LZ1). Blue line: theoretical distribution (*T*); red line: observed distribution (*O*). Orange markers on (**a**) and orange spheres on (**b**) denote catalytic residues (E35, D53). Green segments on (**a**,**b**) span residue range 55–58.

In addition to the drastic example of the incompatibility of the hydrophobicity distribution versus the idealized distribution, human lysozyme is an example of a protein (PDB ID: 1LZ1) with clearly marked fragments with the distribution *O* and *T* highly compatible with the present chain segments showing local incompatibility (Figure 7).

The region of incompatibility is located in the active center of this enzyme, as shown by the compared *T* and *O* profiles of this protein. The RD value for lysozyme is 0.529. The elimination of the catalytic residues (35 and 53) from the status calculations (according to divergence entropy) results in the reduction of the RD value to 0.515. After further elimination of residues 55–58, the value RD = 0.499 was obtained. It should be noted that residues 55–58 are located in the immediate vicinity of the active site and are probably an integral component of the active site (Figure 7).

The elimination of the indicated residues (catalytic and a segment in the immediate vicinity of the catalytic residues) resulting in an RD value less than 0.5 indicates that the entire remainder of the chain satisfies the micelle-like degradation conditions. It also means that the remainder of the chain guarantees the solubility of the protein in question.

**Figure 8:**
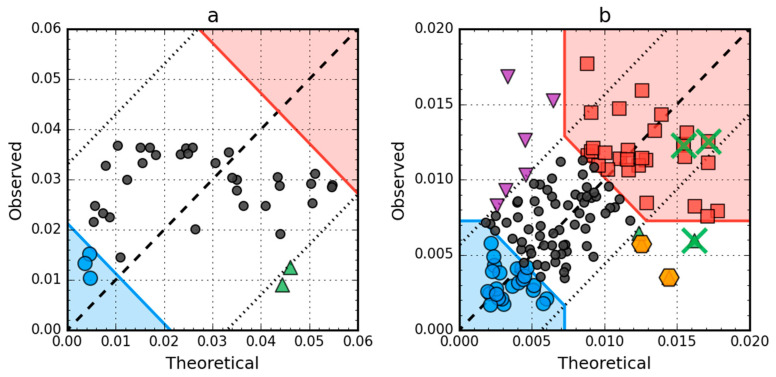
Hydrophobicity density scatter plots (*X*-axis: *T*, *Y*-axis: *O*) of antifreeze protein from winter flounder (PDB ID: 1WFA) (**a**) and human lysozyme (PDB ID: 1LZ1) (**b**). Red square markers: hydrophobic core members; blue circle markers: hydrophilic surface members; green triangle markers: *T* value >> *O* value; purple triangle markers: *T* value << *O* value; black circle markers: hydrophobically insignificant residues. Red and blue backgrounds are areas in which residues are classified as belonging to either core or surface. Orange hexagonal markers on (**b**) denote catalytic residues (E35, D53). Green “×” markers on (**b**) span residue range 55–58 (F57 has been omitted for clarity; it is to the right of the *X*-axis limit, *T* = 0.209).

*T* versus *O* plots of these two proteins (Figure 8) corroborate the above findings. There are no hydrophobic core members in the antifreeze protein (red zone in Figure 8a) and the location of residues in the *T* × *O* space suggests very low correlation between the distributions, which is in fact approximately equal to 0.08. In the lysozyme, catalytic residues are marked by the FOD model as showing hydrophobicity deficiency in the center of the molecule, just like the F57 and Q58, which contribute to the RD value being higher than 0.5. Their elimination (together with the rest of the 55–58 segment) is enough to offset the other increase of RD above 0.5 caused by three exposed cys residues (numbers 6, 77, 128), which can be seen as the top-most triangle markers (purple) on Figure 8b.

## 3. Discussion

Proteins with high RD are mainly proteins representing tertiary structures, where the mutual spatial arrangement of the domains results in the appearance of areas with high RD values. Very often in a protein with the status expressed by the value of RD > 0.5, it is enough to eliminate single residues or small fragments of the chain to obtain the value of RD < 0.5. The residues identified in this way often turn out to be catalytic residues or residues involved in the complexation of another protein. This issue is discussed in the book [42] with a detailed analysis of examples.

For the topic discussed in this article, it is also important to demonstrate the highly ordered hydrophobic core observed in titin [43]. This molecule, used in the SM-FRET method [21] as a frame for the analysis of the protein unfolding process, has a single-step unfolding form. Therefore, easy identification of the unfolding of the frame for other proteins enables a detailed analysis of the unfolding process of other proteins with more complex kinetics. The low RD value for the sandwich domain observed in the immunoglobulin domains just in titin distinguishes it from many other immunoglobulin-like domains [44]. The specificity of the incompatibility of the *T* and *O* distributions in other immunoglobulin-like domains assigns them their specificity.

The early-stage intermediate structure, based on the relaxed arrangement of peptide bond planes, reveals the degree of presence of a seed with a hydrophobic core. The presence of local hydrophobicity maxima compatible with those expected in the structure generated on the basis of Phi_e_ and Psi_e_ angles(and thus partially unfolded) means that the further folding process will be an expansion of the hydrophobic core. Protein folding simulations taking into account the influence of the water environment in the form of introducing an external force field directing the folding process consistent with 3D Gauss function were carried out [45]. A large-scale protein folding project taking into account these external conditions is currently being implemented.

The widely discussed phenomenon of cooperativity in protein folding seems to have its mathematical expression [45]. Generation of the hydrophobic core requires cooperativity and is the effect of a specific synergy, which is obtained in the process of folding in a way strongly dependent on the amino acid sequence. For a given polypeptide chain, the degree of ease/difficulty to obtain this synergy is primarily expressed by the folding time. This ease is obviously determined by the amino-acid sequence, which favors the micelle-like structure more or less. The proteins discussed here are present in hyperthermophilic bacteria. The stable arrangement form of the hydrophobic core is probably the reason for protein stability in these organisms.

The entire non-redundant PDB database showed that a significant number of domains (over 90%) have a hydrophobicity arrangement consistent with the 3D Gaussian distribution [46]. This is in strict accordance with the reports on two-state systems present in protein domains as structural units [47].

The same is true of antifreeze type III proteins, which show hydrophobicity distribution consistent with the 3D Gauss distribution [48]. Interpretation of this phenomenon explains their antifreeze action relying on the arrangement of water molecules in a way resulting from the distribution of charges on the surface of this protein, which is different from those present in the ice structure.

Noteworthy is the de novo protein synthesis experiment, the aim of which was to obtain a significant structural change with a minimum number of introduced mutations. It was obtained for a set of four proteins (56 aa), which differ in turn by one introduced mutation, resulting in alternating structures: 2LHC—3α, 2LHD—4β + α, 2LHG 3α and 2LHE 4β + α [49]. The analysis carried out on the basis of the fuzzy oil drop model showed that the cause of the different secondary structure involved different chain fragments in the construction of the hydrophobic core [37].

What do we learn from this paper that we did not know before?

1. Protein folding is the process of micellization of bipolar molecules (amino acids are molecules with different forms of bipolarity) with limited freedom of movement resulting from the presence of peptide bonds [42].
2. Down-hill proteins (fast-folding, ultrafast-folding) are proteins with an amino acid sequence that allows construction of a micelle-like structure, which in the case of antifreeze proteins guarantees solubility (i.e., the basic condition enabling the performance of a biological function, which is the order of water molecules imposed by the distribution of charges on the protein surface preventing the structuring present in the ice structure). This issue is discussed in this paper.
3. Amino acid sequence determining the protein structure is a sequence with a specific coding system excluding the possibility of generating the perfect micelle, which is the local discordance versus 3D Gauss. The discordance is represented as local hydrophobicity deficiency and usually appears in the ligand or substrate binding cavity [32] or local hydrophobicity excess which is “used” by proteins to construct interfaces in protein–protein complexes [31]. Lysozyme is an example of cavity identification as shown in this paper.
4. The form and degree of the encoded inability to reconstruct the micelle-like pattern determines the specificity of a given protein.
5. In this context, amyloid (formed in physiological conditions) is also a result of micellization. The final product however takes the form of ribbon-like micelle since these polypeptides are not able to construct the globular forms [50].

The above mentioned conclusions are the generalizations resulting from the current work but also from the analysis of numerous proteins described in other publications [33,34,35,36,42,43,50]. The present work discusses a set of proteins representing a high agreement of hydrophobicity distribution in relation to an idealized distribution consistent with 3D Gauss function. In summary, proteins can be defined as “intelligent micelles” with encoded local discordance which expresses the specificity of particular protein molecules. “Amino acid sequence determines the structure” can be substituted by, “amino acid sequence determines the form and degree of specific disability in forming the ideal micelle.” This disability determines the specificity of the protein.

## 4. Materials and Methods

### 4.1. Data

The object of analysis are the proteins listed in Table 2. They represent examples of proteins classified as fast folding. Proteins except de novo, viral and ones derived from Homo sapiens are bacterial proteins.

The list of proteins that are the subject of analysis also includes a negative example in the sense of being assessed by the fuzzy oil drop model. It is a protein of small size (37 aa) taking a helical form along the entire chain length. This form, not representing the globular form, does not necessarily generate the presence of a hydrophobic core.

### 4.2. Late Stage Model—Fuzzy Oil Drop

The fuzzy oil drop model (FOD) has already been described in detail [59,60,61]. The oil drop model introduced by Kauzmann [62] was modified to form the fuzzy oil drop model. There are two levels in this model, which are high in the center of the molecule and low in the surface layer. These have been modified to a continuous form by introducing a 3D Gaussian function spread over the protein molecule. In this situation, each amino acid can be assigned the so-called *T_i_*, which is the level of hydrophobicity resulting from the location of a given residue represented by the effective atom (averaged position of atoms making up the amino acid). This is the so-called idealized level (i.e., the level that a given residue would represent if the distribution was completely compatible with the theoretical distribution (*T*)).

In order to determine the status consistent with the 3D Gaussian distribution, the following function was used:

(1) $${\tilde{H}}_{i}^{T}=\frac{1}{H_{\mathrm{sum}}^{T}}\exp\left(\frac{-{\left(x_{i}-\bar{x}\right)}^{2}}{2\sigma_{x}^{2}}\right)\exp\left(\frac{-{\left(y_{i}-\bar{y}\right)}^{2}}{2\sigma_{y}^{2}}\right)\exp\left(\frac{-{\left(z_{i}-\bar{z}\right)}^{2}}{2\sigma_{z}^{2}}\right).$$

The point $\left(\bar{x},\bar{y},\bar{z}\right)$ is the position of the geometric center of the protein in the 3D coordinate system, placed in its origin, at (0,0,0). The protein should be oriented, making the line linking the longest distance between two effective atoms in the molecule coaxial with the *X*-axis. It is then rotated around the *X*-axis to make the line linking the two most distant positions of the projections of effective atoms on the YZ plane coaxial with the *Y*-axis. The three parameters *σ_x_*, *σ_y_*, *σ_z_* represent standard deviations of the size of the protein, equal to 1/3 of the highest absolute values of the x-coordinate, y-coordinate and z-coordinate, respectively (according to the 3-sigma rule). The normalizing coefficient $H_{\mathrm{sum}}^{T}$ represents the sum of all $H_{i}^{T}$ values of amino acids of the protein, making the $H_{i}^{T}$ value unit-less. The only input information for the theoretical distribution is a geometrical term concerning the full protein (i.e., the size of the ellipsoid “drop” containing the protein, characterized by *σ_x_*, *σ_y_*, *σ_z_*). Traditionally, the value of the Gauss function is interpreted as a theoretical idealized hydrophobicity density at any given point.

However, the status of a given amino acid may not be as expected. The actual status is the effect of the magnitude of the interactions of a given residue with neighboring residues. Thus, it depends on the distance between the interacting residues and on their own hydrophobicity. The observed level of hydrophobicity, called O, characterizes the status of a given residue in a given protein.

The equation proposed in [63] was applied to calculate the observed hydrophobicity:

(2) $$H_{i}^{0}=\frac{1}{H_{sum}^{0}}\underset{j}{\sum }\{\begin{array}{c} \left(H_{i}^{r}+H_{j}^{r}\right)\left(1-\frac{1}{2}\left(7{\left(\frac{r_{ij}}{c}\right)}^{2}-9{\left(\frac{r_{ij}}{c}\right)}^{4}+5{\left(\frac{r_{ij}}{c}\right)}^{6}-{\left(\frac{r_{ij}}{c}\right)}^{8}\right)\right),\;for\;r_{ij}\leq c,\\ 0,forr_{ij}>c \end{array}$$

where $H_{i}^{O}$ denotes the experimentally observed hydrophobic density at a particular point which collects the hydrophobic interaction in distance-dependent form, as given in the formula with the cutoff distance (*c*) assumed according to original work 9Å. The denominator $H_{\mathrm{sum}}^{O}$ (sum of all $H_{i}^{O}$) makes the value in normalized form. $H_{i}^{r}$ and $H_{j}^{r}$ express the intrinsic hydrophobicity of *i*-th and *j*-th residues, which can be taken according to the arbitrarily selected scale.

After normalizing distributions *T* and *O*, it becomes possible to compare them quantitatively using divergence entropy $D_{\mathrm{KL}}$ introduced by Kullback-Leibler [64].

(3) $$D_{KL}(P||Q)=\sum_{i}P\left(i\right)\log_{2}\frac{P\left(i\right)}{Q\left(i\right)},$$

where *P*(*i*) denotes the observed probability (hydrophobicity density) localized on the *i*-th residue, which in this paper is called *O_i_* and *Q*(*i*) denotes the expected (target distribution) hydrophobicity localized on the same residue, which in this paper is called *T_i_* (theoretical one) corresponds to the distance between *O* and *T*, the latter of which is regarded as the reference.

Equation (3) takes the following form in our paper:

(4) $$D_{KL}(O|T)=\sum_{i=1}^{N}O_{i}\log_{2}\left(O_{i}/T_{i}\right),$$

with *T_i_* treated as reference distribution.

*D_KL_* expresses the formal distance between both distributions (*T* and *O*). However, since it constitutes a measure of entropy, it cannot be interpreted on its own and a second reference model must be provided. Since *T* simulates a “perfect” hydrophobic core, we may add a reference distribution which lacks any concentration of hydrophobicity at any point in the protein body. This type of distribution—called the unified distribution (*R*)—assigns hydrophobicity of 1/N to each residue (N being the number of residues in the chain). It represents the status deprived of any form of hydrophobicity differentiation in the protein body.

(5) $$D_{KL}(O|R)=\sum_{i=1}^{N}O_{i}\log_{2}\left(O_{i}/R_{i}\right),$$

with *R_i_* as the reference distribution.

When considering *O* and *R*, the value of *D_KL_* tells us to what degree the observed distribution approximates the unified distribution. Comparing both values (for *O*/*T* and *O*/*R*) provides a description of the protein’s status. When *O*/*T* < *O*/*R*, the observed distribution is aligned with the theoretical distribution, and therefore the protein may be assumed to contain a hydrophobic core. In the opposite case (*O*/*T* > *O*/*R*) the protein lacks a prominent core.

By applying the 3D Gaussian model and calculating divergence entropy, we obtain a fine-grained description of the protein’s status, a procedure which would not be possible under the original oil drop model. In order to avoid having to deal with two distinct values of *D_KL_*, we compute another parameter referred to as relative distance (RD):

(6) $$\mathrm{RD}=\frac{D_{KL}(O|T)}{D_{KL}(O|T){+D}_{KL}(O|R)}.$$

The parameter RD is used to determine the status of a given molecule or domain. When assuming values less than 0.5, this indicates an arrangement consistent with the idealized system. Otherwise, the situation differs from the centric system of the hydrophobic core. The status determined may refer to the whole protein or to a domain and an appropriate 3D Gaussian function is generated for such a defined structural unit. It is also possible to determine the status of a selected chain fragment within a given unit (protein, domain), or to identify those residues which together represent a status deviating from the centric arrangement. Such a calculation is used, for example, to determine the status of the segment forming a disulfide bond, the surroundings of the catalytic residue, or the status of the residues involved in ligand binding. This procedure has been used in the analysis discussed here.

The RD parameter can be calculated for any structural unit under consideration. The status of the selected chain fragment can also be estimated. The status of the chain fragment can be treated only as part of a formerly defined structural unit. The status of a chain fragment requires normalization (sum of *T_i_* and *O_i_* belonging to selected chain fragments shall be equal to 1.0). Then the RD can be calculated after defining the structural unit it has been selected from. It may happen that the status of selected a chain fragment can be expressed by RD > 0.5 in respect to the chain or the domain of the status expressed by RD < 0.5.

### 4.3. Early-Stage Intermediate Model—Partially Unfolded Protein

When looking for sources of generating clusters of hydrophobic residues in the center of the molecule, an early-stage intermediate model was used for the analyzed proteins. It should be noted that it was constructed based solely on the preferences of the backbone itself. The system of peptide bonds—including the system of peptide bond planes in particular—showed that the relaxed forms of these planes determine the relationship of Phi and Psi rotation values and the radius of curvature for the pentapeptide is treated as a unit. The value of the angle between the planes of peptide bonds (with the virtual Cα-Cα bond as the axis of rotation) determines the value of the radius of curvature. Following angle changes from 0° to 180°, the radius of curvature changes from the smallest present in the helix to the largest present in the extender or beta form. These changes, determined with the use of the approximation function, indicate on the Ramachandran map an elliptical path connecting all areas with a specific secondary structure. It is assumed that as long as the backbone alone determines the structural form, we are dealing with an early state of the folding process. Replacing the Phi and Psi angles with the corresponding Phi_e_ and Psi_e_ angles (angles belonging to the elliptical path determined by the shortest distance between Phi and Psi and the ellipse) generates the form of an early intermediate when inter amino acid interactions do not take place yet.

Early intermediate structures are obtained using the Phi_e_ and Psi_e_ values. Such an analysis leads to the recognition of the 3D structure, in which—if the non-binding interactions present in proteins are included in the calculations—the final form is obtained.

The simulation of the folding process, where the starting structure is determined precisely on the basis of the described model and the folding process relies on minimizing non-binding interactions and taking into account the active participation of the water environment by introducing an external force field in the form of a 3D Gaussian function, has been presented [65].

Here, the early intermediate structures determined for the proteins in question will be analyzed comparatively in order to identify a common factor leading to downhill or fast-folding structures characterized by a high degree of agreement between the *T* and *O* distribution. This observation is interpreted as an example of the folding process dominated by a tendency to the generation of a hydrophobic core. The unequivocal orientation of the folding process as dominated by the environment explains the speed of the folding process and its reversibility. The distribution of the Phi_e_ and Psi_e_ angles obtained for the non-redundant protein database shows the presence of seven local maxima who have been assigned codes A through G. The C code represents the helical form, the G code is the left-handed helix, the E code is the beta structure, and the F code represents the structure of the end of beta structure segment. The interesting state is represented by the D code that connects the helical region to the beta structure region.

The early intermediate structures of the proteins in question will be expressed in the codes given above. With the help of the determined Phi_e_ and Psi_e_ values, it is possible to construct a partially unfolded structure determined due to the optimal arrangement of the planes of peptide bonds.

### 4.4. Tools and Software

3D images of the protein structures were rendered with PyMOL [66] while data charts were plotted using Matplotlib library [67]. Online calculations of fuzzy oil drop hydrophobicity profiles and structural codes are available at http://fod.cm-uj.krakow.pl web server.

## 5. Conclusions

In the present study, significant participation of the arrangement of hydrophobicity distribution compatible with that present in the spherical micelle and expressed by means of 3D Gauss function in fast and ultrafast folding as well as downhill proteins were demonstrated [68]. The reversibility of this process results from the presence of the seed of the hydrophobic core in the early intermediate structure, which is the partially folded structure. In the present work, the participation of hydrophobic interactions in the process of shaping the structure of proteins has been expressed by means of a mathematical model [42,69]. The fuzzy oil drop model is nothing more than the mathematical expression of the commonly observed hydrophobic core generation. The model allows quantitative description and comparison of the status of different protein molecules.

The practical use of the fuzzy oil drop model allows for the quantitative assessment of the status of a given protein, enabling comparative analysis. The higher the RD value, the lower the stability guaranteed by the presence of the hydrophobic core. For proteins with low RD values, a one-step unfolding process is predicted. The low value of RD observed for most domains treated as individual structural units (3D Gaussian function determined for the domain) suggests their spontaneous folding as an expression of the influence of the external field, which is the water environment, directing this process towards the generation of a hydrophobic core.

## Figures and Tables

**Table 1:** The values of the RD (Relative Distance) parameter for proteins under study.

PDB ID	RD—LS	RD—LS after Fragment Selection/Elimination		RD—ES	RD—ES after Fragment Selection/Elimination	
**2CYU**	**0.638**	3-helix system	0.493	**0.540**	18–23	0.431
**2L6Q**	0.473			**0.595**	7–10,21–26	0.488
**2L6R**	0.477	34–37 eliminated	0.462	0.439		
**1BBL**	**0.556**	12–33 selected	0.349			
		38–48 selected	0.311			
**2WXC**	0.382			**0.525**	165-C-term	0.441
**2A3D**	**0.580**	21–24 eliminated	0.474	**0.710**	28–45 selected	0.474
**2F21**	0.402			**0.761**		
		1–49 selected	0.520	**0.657**		
		50–163 selected	0.310	**0.697**		
**2P6J**	0.298			**0.504**	helix 5–17	

ES (early, limited conformational sub-space) and LS (late stage, structure available in PDB) forms, respectively. Selected: status of the given fragment showing the local accordance; Eliminated: the RD value after elimination of a given fragment; Helix orientation: only the re-orientation of a given helix expected for lowering the RD value; Values in bold: structure with RD above 0.5.

**Table 2:** List of proteins under consideration in this paper.

PDB-ID	Protein	Chain Length	Characteristics	Ref
2CYU	e3-binding domain	39 aa	Downhill	[51]
2L6Q	Viral protein	62 aa		[52]
2L6R	Viral protein	62 aa		[53]
1W4E	Peripheral-subunit binding domain	45 aa	Ultrafast	[54]
1W4F		45 aa		[54]
1W4G		45 aa		[54]
1W4H		45 aa		[54]
2BTH		45 aa		[54]
1W4J		51 aa		[54]
1W4K		51 aa		[54]
1BBL		37 aa		[55]
2WXC		47 aa		[56]
Negative Control				
1WFA	Antifreeze	37 aa	Antifreeze	[57]
1LZ1	Lysozyme	130 aa	Enzyme	[58]

## Supplementary Materials

Supplementary materials can be found at https://www.mdpi.com/1422-0067/21/20/7632/s1. Figure S1. Hydrophobicity density distributions and 3D presentation of native (**a**,**c**,**e**) and early stage (**b**,**d**,**f**) forms of de novo designed triple helix bundle protein (PDB ID: 2A3D); Figure S2. Hydrophobicity density distributions and 3D presentation of native (**a**,**c**,**e**) and early stage (**b**,**d**,**f**) forms of engrailed homeodomain (PDB ID: 2P6J).

## Abbreviations

FOD	Fuzzy oil drop
PDB	Protein data bank
RD	Relative distance
