# Supplementary material for: Downhill, Ultrafast and Fast Folding Proteins Revised
Source: Int J Mol Sci. 2020 Oct 15;21(20):7632. doi: 10.3390/ijms21207632 (PMC7589632; doi:10.3390/ijms21207632)
Supplement: Supplementary file 1 [file ijms-21-07632-s001.pdf]

International Journal of
Molecular Sciences

MDPI

Supplementary materials

Downhill, Ultrafast and Fast Folding Proteins Revised

Mateusz Banach [1], Katarzyna Stapor [2], Leszek Konieczny [3], Piotr Fabian [2] and Irena Roterman [1,*]

[1] Department of Bioinformatics and Telemedicine, Jagiellonian University—Medical College, Lazarza 16, 31-533 Krakow, Poland; mateusz.banach@uj.edu.pl (M.B.); myroterm@cyf-kr.edu.pl (I.R.)

[2] Institute of Computer Science, Silesian University of Technology, Akademicka 16, 44-100 Gliwice, Poland piotr.fabian@polsl.pl (K.S.); katarzyna.stapor@polsl.pl (P.F.)

[3] Chair of Medical Biochemistry, Medical College, Jagiellonian University, Kopernika 7, 31-034 Krakow, Poland; mbkoniec@cyf-kr.edu.pl

* Correspondence: myroterm@cyf-kr.edu.pl; Tel.: +48-12-619-96-93

Received: 30 August 2020; Accepted: 11 October 2020; Published: date

S1. De novo designed triple helix bundle protein (2A3D)

The de novo designed triple helix bundle protein (PDB ID: 2A3D) (Figure S1) presents an RD > 0.5. The elimination of only four residues from loops 21–24 (Fig. S1a) results in a significant decrease in the value of RD, suggesting that almost the entire system shows the micellar arrangement. Partially unfolded status is shown by the presence of the seed of the core formed by helix 54–71 and partly by the helices 1–21 and 23–27 (Figure S1b). Only the 28–45 helix shows a pattern different from that expected for the generation of the hydrophobic core in the early intermediate structure (Figure S1b). The presence of the seed of the hydrophobic core in this protein in a partially unfolded form suggests the possibility of its production in the further stages of the folding process.

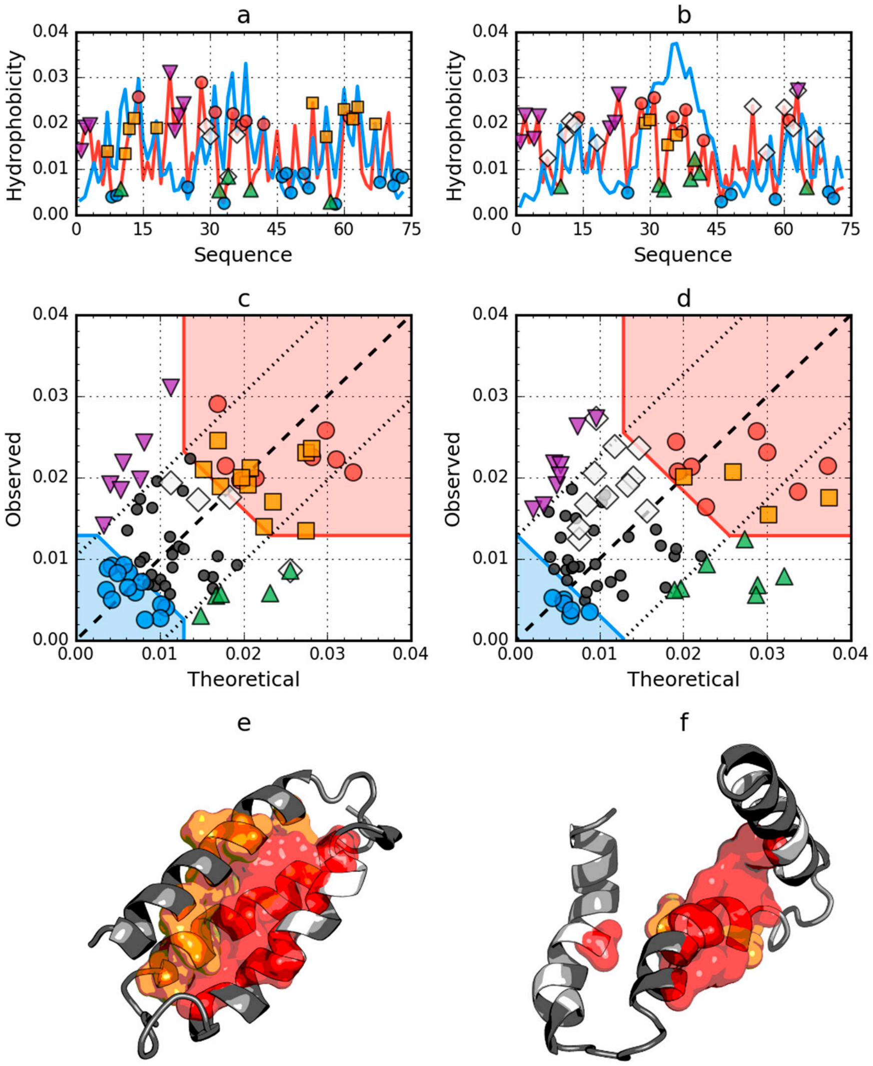

Figure S1. Hydrophobicity density distributions and 3D presentation of native (**a**,**c**,**e**) and early stage (**b**,**d**,**f**) forms of de novo designed triple helix bundle protein (PDB ID: 2A3D). Blue line on (**a**) and (**b**) – theoretical distribution (*T*); red line on (**a**) and (**b**) – observed distribution (*O*). Red zone on (**c**) and (**d**) – hydrophobic core; blue zone on (**c**) and (**d**) – hydrophilic surface. Red circle markers – common hydrophobic core members in both models. Orange square markers – hydrophobic core members in this model but not in the other model. White rhombus markers – hydrophobic core members in other model but not in this model (possible overlap with other markers). Blue circle markers – hydrophilic surface members in this model. Green triangle markers – *T* value >> *O* value; Purple triangle markers – *T* value << *O* value. Black circle markers – hydrophobically insignificant residues. Colored segments on (**e**) and (**f**) correspond to markers of the same color on (**a**), (**c**) and (**b**), (**d**), respectively (hydrophobic core is shown as surface).

S2. Thermostable protein variant- engrailed homeodomain (2P6J)

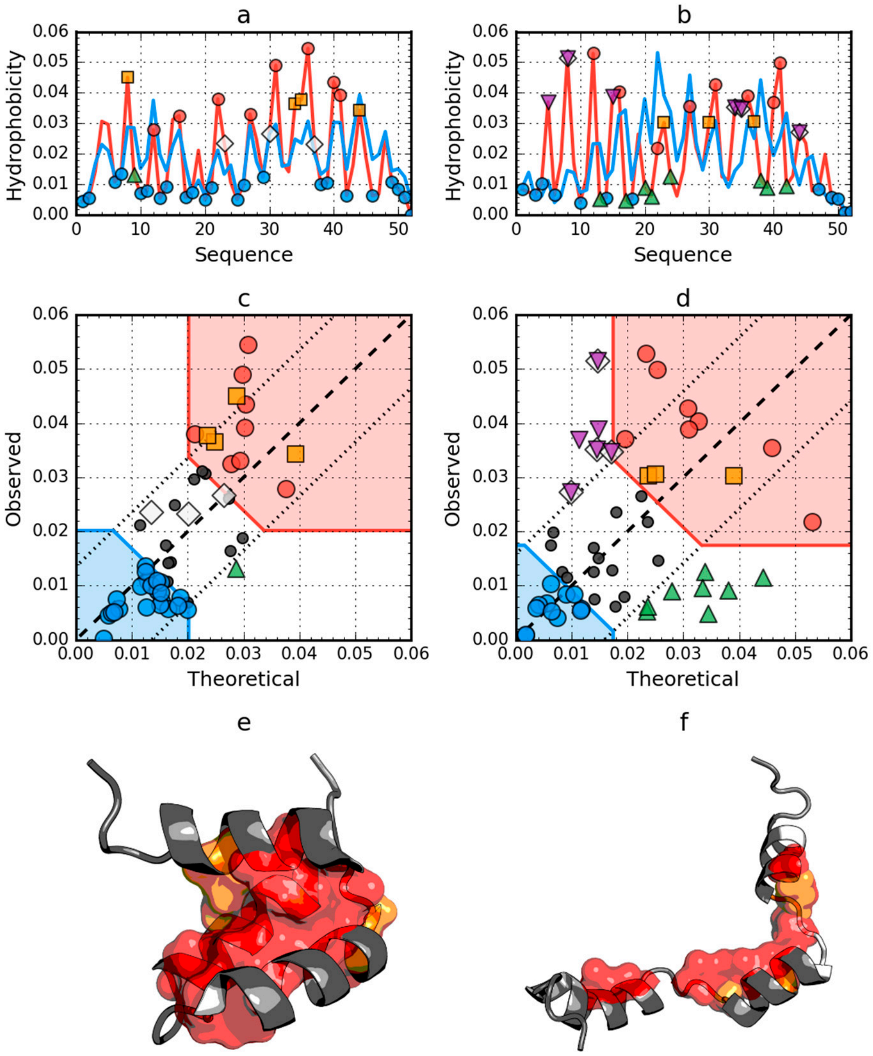

Figure S2. Hydrophobicity density distributions and 3D presentation of native (**a**,**c**,**e**) and early stage (**b**,**d**,**f**) forms of engrailed homeodomain (PDB ID: 2P6J). Blue line on (**a**) and (**b**) – theoretical distribution (*T*); red line on (**a**) and (**b**) – observed distribution (*O*). Red zone on (**c**) and (**d**) – hydrophobic core; blue zone on (**c**) and (**d**) – hydrophilic surface. Red circle markers – common hydrophobic core members in both models. Orange square markers – hydrophobic core members in this model but not in the other model. White rhombus markers – hydrophobic core members in other model but not in this model (possible overlap with other markers). Blue circle markers – hydrophilic surface members in this model. Green triangle markers – *T* value >> *O* value; Purple triangle markers – *T* value << *O* value. Black circle markers – hydrophobically insignificant residues. Colored segments on (**e**) and (**f**) correspond to markers of the same color on (**a**), (**c**) and (**b**), (**d**), respectively (hydrophobic core is shown as surface).

The engrailed homeodomain (PDB ID: 2P6J) shows highest degree of compatibility with the fuzzy oil drop model among native protein forms in this paper (RD = 0.298) and its partially unfolded form shows a minimally exceeded level of 0.5 for the RD parameter (Figure S2b). Eliminating a single residue (e.g. 39) improves the RD value to less than 0.5. The distribution profile of T and O indicates that it is enough to reorient helix 5–17 and the hydrophobic core is restored (Figure S2a).
